# Design and Synthesis of Bis-amide and Hydrazide-containing Derivatives of Malonic Acid as Potential HIV-1 Integrase Inhibitors

**DOI:** 10.3390/molecules13102442

**Published:** 2008-10-01

**Authors:** Mario Sechi, Ugo Azzena, Maria Paola Delussu, Roberto Dallocchio, Alessandro Dessì, Alessia Cosseddu, Nicolino Pala, Nouri Neamati

**Affiliations:** 1 Dipartimento Farmaco Chimico Tossicologico, Università di Sassari, Via Muroni 23/A, 07100 Sassari, Italy; E-mails: paoladelussu@libero.it (M-P. D.); nikpal@uniss.it (N. P.); 2 Dipartimento di Chimica, Università di Sassari, Via Vienna 2, 07100 Sassari, Italy; E-mail: ugo@uniss.it; 3 CNR-Istituto di Chimica Biomolecolare, Sassari, 07040 Li Punti, Italy; E-mails: roberto.dallocchio@icb.cnr.it (R. D.); alessandro.dessi@icb.cnr.it (A. D.); 4 Dipartimento Farmaco Chimico Tecnologico, Università di Siena, Via A. Moro, 53100 Siena, Italy; E-mail: alessia.k@libero.it; 5 Department of Pharmacology and Pharmaceutical Sciences, University of Southern California, School of Pharmacy, 1985 Zonal Avenue, PSC 304, Los Angeles, California, 90089, USA

**Keywords:** Bis-amides, Hydrazides, Malonic acid, HIV-1, HIV-1 integrase, Docking studies.

## Abstract

HIV-1 integrase (IN) is an attractive and validated target for the development of novel therapeutics against AIDS. In the search for new IN inhibitors, we designed and synthesized three series of bis-amide and hydrazide-containing derivatives of malonic acid. We performed a docking study to investigate the potential interactions of the title compounds with essential amino acids on the IN active site.

## Introduction

Therapeutic protocols for the treatment of HIV infection are mainly based on the combined use of reverse transcriptase, protease, and more recently, of cell fusion and entry inhibitors. Although drugs targeting reverse transcriptase and protease are in wide use and have shown effectiveness, the rapid emergence of resistant variants, often cross-resistant to the members of a given class, limits the efficacy of existing antiretroviral drugs. Therefore, it is critical to develop new agents directed against alternate sites in the viral life cycle.

In this context, HIV-1 integrase (IN), the enzyme that mediates an obligatory step in the viral replication process by catalyzing the integration of viral cDNA into the host genome, represents a validated target for the development of new drugs against HIV-1 infection [1]. Because IN does not have a human homologue, it represents one of the most promising targets in AIDS research.

**Figure 1:**
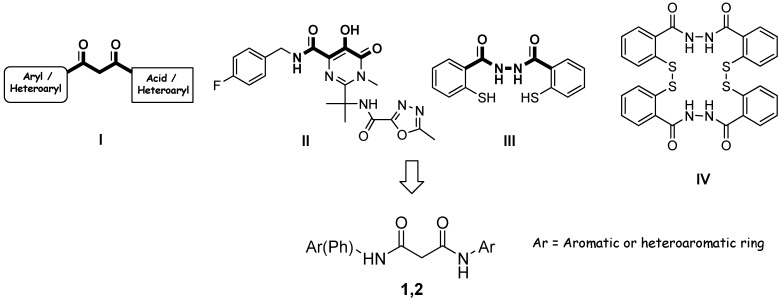
Design of the title compounds.

In the past several years, numerous compounds with diverse structural features have been reported as IN inhibitors [2, 3]. In particular a number of compounds bearing a β-diketo acid moiety (DKA, **I**, Figure 1) were discovered as new selective and potent inhibitors [4], and some of them have emerged as the most promising lead in anti-HIV-1 IN drug discovery.

Recently, the IN inhibitor MK-0518 (Merck & Co., Inc., **II**, Figure 1), a DKA-like compound, has been approved in therapy [5]. In the light of these promising results it is important to design new and potent IN inhibitors.

**Figure 2:**
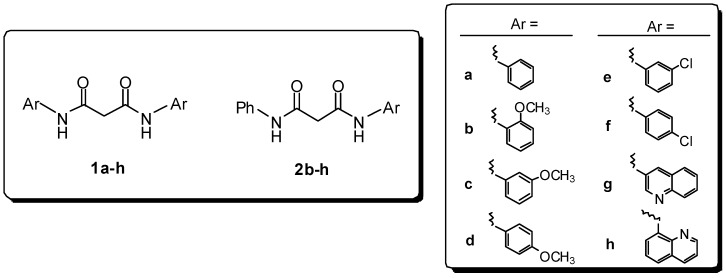
Symmetrical and asymmetrical bis-amides **1** and **2**.

To identify novel and unified pharmacophore required for activity we selected and formally combined the main structural motifs of **I** and **II** together to the hydrazide functionality of compounds **III** and **IV** (Figure 1), previously reported as a new class of selective IN inhibitors having antiviral activity [6]. With this in mind, we designed two sets of symmetrical (**1**) and asymmetrical (**2**) bis-amide derivatives of malonic acid (Figure 1 and Figure 2).

**Figure 3:**
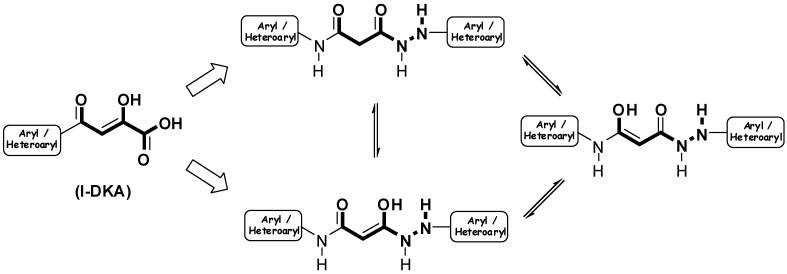
Design of hydrazides **3**.

Although several synthetic and biological studies on DKAs and DKA-based compounds have been reported [1, 4], the mechanism responsible for inhibition of the IN still remains uncertain. It is believed that the β-diketo acid pharmacophoric motif could be involved in a functional sequestration of one or both divalent metal ions in the enzyme catalytic site, to form a ligand-M^2+^-IN complex, which blocks the formation of the IN-DNA complex by competing with the target DNA substrate [1]. In light of such hypothesis we focused on structures bearing a tautomeric moiety, as in **I**-DKA (Figure 3), with the aim that the cation coordination at the IN catalytic site is favoured. The possibility to obtain a pharmacophoric fragment suitable to generate tautomers and complexes with metal ions was supported by the previoulsy reported behaviour of β-ketoamide and hydrazide systems [7,8,9,10,11].

**Figure 4:**
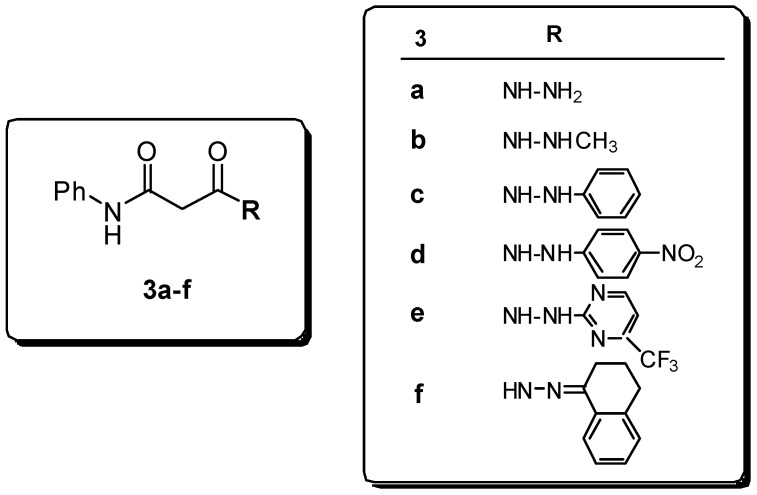
Hydrazides **3**.

On this basis, a new set of molecules with structures **3a-f** (Figure 4) was designed and synthesized considering a) bulkiness of substituents, and b) the substitution of an amidic motif with a hydrazidic one, hopefully suitable for coordination with the enzyme catalytic site and/or with metallic cofactors.

## Results and Discussion

### Preparation of bis-amides.

**Scheme 1:**
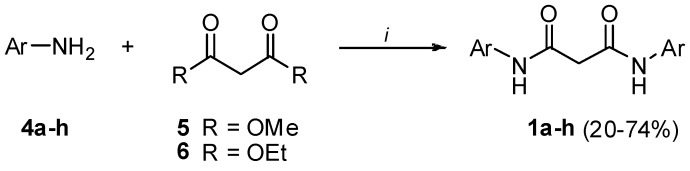
Preparation of bis-amides **1a-h**.

For the synthesis of symmetrical and asymmetrical bis-amide derivatives of malonic acid different approaches were followed. The symmetric amides **1a-h** were synthesized in agreement with the procedure reported by Vennerstrom and Holmes for aminolysis of methyl and ethyl malonic esters [12] (Scheme 1).

*Reagents and conditions*: *i*) reflux, 3.5-5 h.

**Scheme 2:**
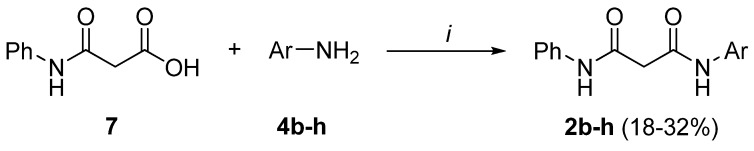
Preparation of bis-amides **2b-h**.

Asymmetric bis-amides **2b-h** were obtained in 18-32 % yields by reacting monophenyl amide **7** with the appropriate arylamine in the presence of 4-(4,6-dimethoxy-[1,3,5]-triazyn-2-yl)-4-methyl-morpholine chloride (DMTMM) [13, 14] (Scheme 2).

*Reagents and conditions*: *i*) DMTMM, THF, r.t., 24 h.

**Scheme 3:**
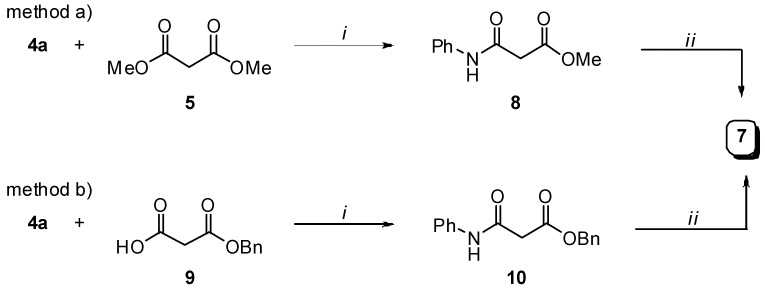
Preparation of intermediate **7**.

Desymmetrization of dimethyl ester **5** to monoamide **7** was accomplished via aminolysis with one equivalent of aniline **4a** to give **8**, which was separated by filtration and readily hydrolized with two equivalents of Na_2_CO_3_ in water (Scheme 3, method a) [15]. Alternatively, treatment of the commercially available monobenzyl ester **9** with aniline **4a** in the presence of DMTMM afforded **10** that was converted to the desired **7** by hydrogenolysis at r.t. (Scheme 3, method b). The use of DMTMM has been reported to be particularly useful for the formation of amides starting from carboxylic acids and aromatic amines [16].

*Reagents and conditions*: Method a: *i*) 190 °C, 30 min; *ii*) H_2_O, Na_2_CO_3_, reflux 30 min; Method b: *i*) DMTMM, THF, r.t., 24 h; *ii*) MeOH, H_2_, 5% Pd/C, 12 h.

### Preparation of hydrazides.

**Scheme 4:**
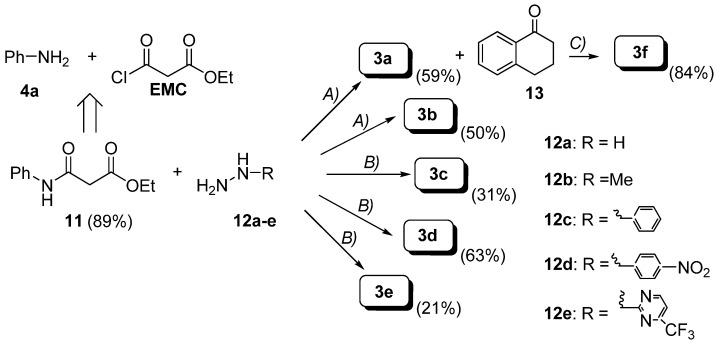
Preparation of hydrazides **3a-f**.

Similar to the afore-mentioned synthetic strategy for bis-amides **1a-h**, hydrazides **3a-e** were synthesized by condensation of the amido ester **11** with the corresponding hydrazine **12a-e** in ethanol [17] or chlorobenzene [18] under reflux, although a slight modification in the procedure for **3f** was required (Scheme 4).

*Reagents and conditions*: A) Ethanol, reflux 4 h for **3a**, 30 h for **3b***.* B) Chlorobenzene, reflux, 20 h for **3c**, 15 h for **3d**, 25 h for **3e**; C) Ethanol, reflux, 5 h. - For **11**: TEA, acetone, r.t., 1 h.

**Scheme 5:**
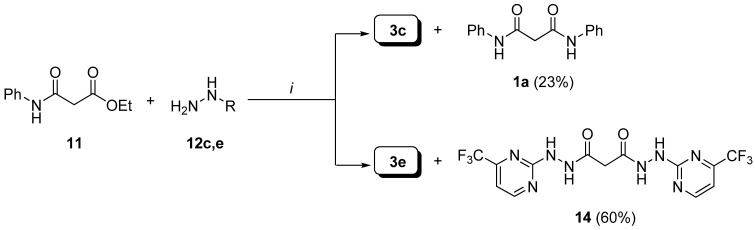
Formation of compounds **1a** and **14**.

Intermediate **11** was prepared according to Ukrainets *et al*. [19] by nucleophilic substitution of aniline **4a** on ethyl malonyl chloride (EMC). As depicted in Scheme 5, condensation of **11** with **12c** afforded the expected **3c** along with the dianilide **1a** (23%), which was characterized by comparison with data reported in the literature [19,20]. Moreover, treatment of **11** with **12e** gave the bis-hydrazide **14** as the major product (**14** = 60% *vs* **3e** = 21%).

*Reagents and conditions*: *i*) Chlorobenzene, reflux, 24 h.

**Figure 5:**
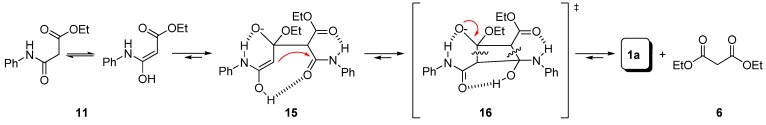
Hypothetical mechanism for the formation of **1a**.

The formation of the bis-anilide **1a** (along the formation of diethylmalonate 6) can be rationalized via an intermolecular condensation of **11** in its tautomeric form to give the adduct **15**, which can cyclize intramolecularly to afford the four membered cyclic transition state **16** followed by structural collapse as end game (Figure 5) [19].

The synthesis of symmetrical bis-anilides **1a**-like has previously been reported. It consists of treatment of diethyl esters of the malonic acids and anilines in solvent free conditions (170-180 °C), that it has been used for the preparation of **1a-h** (as depicted in Scheme 1), or under reflux in high boiling point solvents such as DMF [19]. In the example reported herein, the formation of **1a** is facilitated because **11** (enolic tautomer) can allow the ester carbonyl to be in conjunction with the aniline system causing further reduction of its electronic density and therefore favouring the formation of the adduct **15**.

On the other hand, the new compound **14** is likely to be formed from transamidation of the desired **3e** as a discrete excess of hydrazide **12e** (0.5 eq) is used. Full characterization of **14** was accomplished by NMR, mass spectrometry and elemental analysis. Compound **3f** was obtained in 89% yield by condensation of hydrazide **3a** with commercially available α-tetralone **13** in ethanol under reflux.

Finally, analysis of the ^1^H-NMR and ^13^C-NMR spectra of all described compounds (recorded in DMSO-*d*_6_ or CDCl_3_ + DMSO-*d*_6_ mixture) revealed the presence of the methylene between the two carbonyls, and no extra-OH signals are detected. Moreover, D_2_O exchange involved only amidic NH (**3a-d** and **11**) and hydrazidic NH (**3a-d**). This indicates that such systems seem to exist, in organic solvents, only as diketo tautomers.

### Molecular Modeling

To investigate the putative binding modes of some bis-amides/hydrazides, chosen as model compounds, compared with those of the reference compounds **I-Phe-DKA** [(2*Z*)-2-hydroxy-4-oxo-4-phenylbut-2-enoic acid] and **III**, we performed computational docking studies as described [21,22]. In keeping with several computational docking studies reported in the literature [21,22,23,24,25,26], the IN-5CITEP co-crystal structure (PDB 1QS4) [27] was used.

**Figure 6:**
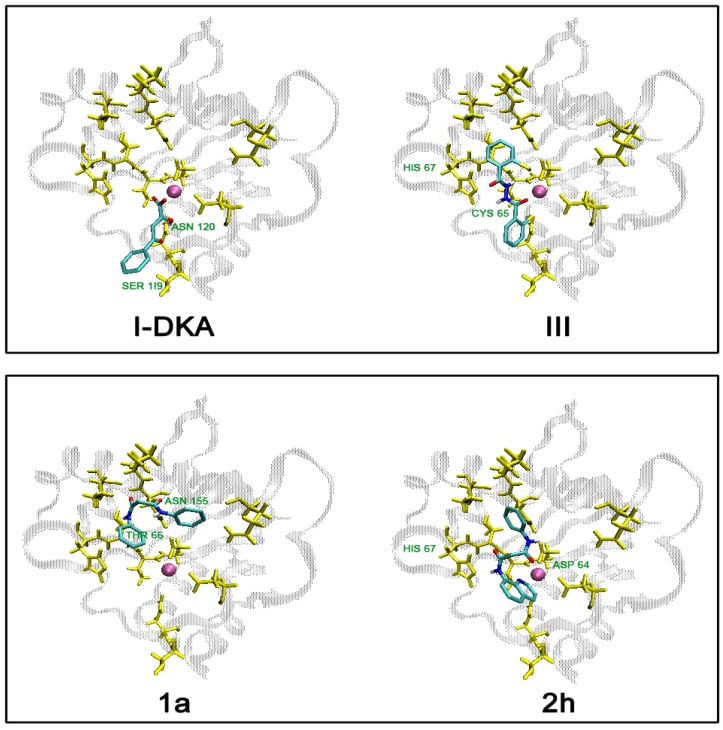
Graphical representation of hypothetical disposition of reference compounds **I-Phe-DKA** and **III**, and representative compounds of symmetrical **1a** and asymmetrical **2h** bis-amides showing the interacting amino acid residues on the HIV-1 IN active site core domain. Mg^2+^ ion is shown in magenta.

**Figure 7:**
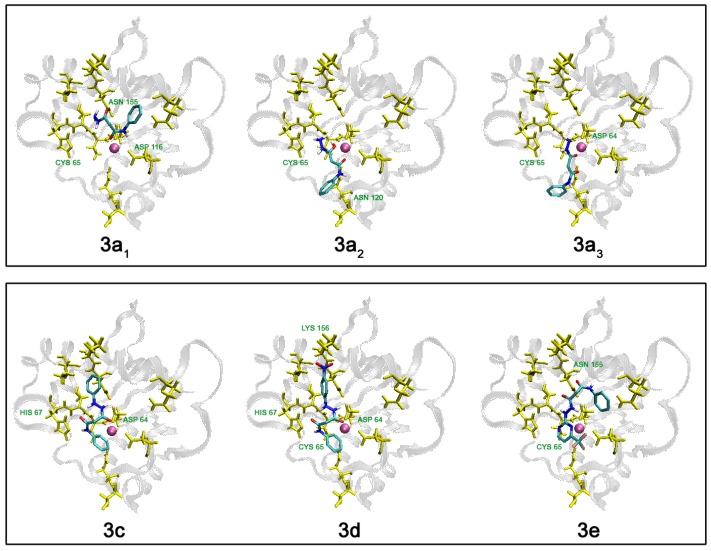
Graphical representation of hypothetical disposition of some tautomers (**3a_1_**, **3a_2_**, **3a_3_**) of the hydrazide **3a**, as well as hydrazides **3c**, **3d**, and **3e**, showing the interacting amino acid residues on the HIV-1 IN active site core domain. Mg^2+^ ion is shown in magenta. 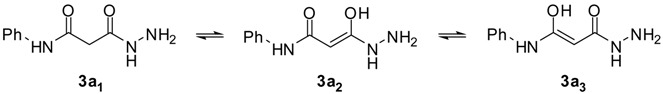

In this study, AutoDock 3.0.5 was used because it utilizes a fully flexible ligand in its docking algorithm (although it is still docked to a rigid protein) and because it has been shown to successfully reproduce many crystal structure complexes [28]. We selected compounds **1a**, **2h**, **3a**, **3c**, **3d**, **3e**, and the reference compounds **I-Phe-DKA** and **III**, and built them in their neutral form (only **I-Phe-DKA** was calculated as carboxylate). Moreover, three putative tautomers (**3a_1-3_**, Figure 7) for the hydrazide **3a** were postulated and included in this preliminary study. The results of clustered docking runs with the most favourable free binding energy are given in Table 1. Graphical representations of top-ranking binding modes obtained for these ligands showing the important residues involved in binding are depicted in Figure 6 and Figure 7.

**Table 1:** Docking results of 100 independent runs for title and reference compounds.

Ligand	*Ntot	^†^focc	°F.D.E.	^‡^∆Gbind	^#^K*i*	H-bonds
**I-Phe-DKA**	35	5/4	-8.94	-7,78	2.00E-06	Ser119, Asn120
**III**	51	8/8	-8.76	-6,78	1.07E-05	Cys65, His67, Glu92
**1a**	39	10/7	-6.65	-5,49	9.38E-05	Thr66, Asn155
**2h**	25	19/9	-8.19	-6,86	9.29E-06	Asp64, His67
**3a_1_**	21	20/14	-5.79	-4,41	5.81E-04	Cys65, Asp116, Asn155
**3a_2_**	50	13/7	-6.62	-5,56	8.34E-05	Cys65, Asn120
**3a_3_**	31	10/7	-6.88	-5,61	7.66E-05	Asp64, Cys65
**3c**	28	14/10	-8.21	-6,55	1.57E-05	Asp64, His67
**3d**	17	26/13	-8.26	-6,56	1.55E-05	Asp64, Cys65, His67, Ly156
**3e**	21	27/12	-7.42	-5,61	7.68E-05	Cys65, Asn155

*Total number of clusters. ^†^Number of distinct conformational clusters found out of 100 runs / number of multi-member conformational clusters. °Final Docked Energy. ^‡^Estimated free binding energy (kcal/mol). ^#^Estimated inhibition constant (M, 298 °K).

According to docking results, the amino acid residues involved in the binding of title compounds located near the catalytic site were as follows: Asp64, Cys65, Thr66, His67, Glu92, Asp116, Ser119, Asn120, Asn155, Ly156. Several of these were considered to be very important for the activity of IN and some have been previously shown to play a role in substrate binding [29,30,23]. In general, different ligands showed different binding modes with some overlapping features, which were predicted as potential *H*-bonds and van der Waals interactions.

The estimated free binding energy values (∆Gbind) of the docked positions, expressed in kcal/mol, indicated favourable interactions and tight binding with key aminoacid residues on the active site of IN. However, with ∆Gbind = -7.78 kcal/mol, **I-Phe-DKA** displayed better energy results than the other compounds, as expected on the basis of its reported potency against IN [31]. High free binding energy values have also been obtained for the reference compound **III**. We also found that **III** binds to Cys65 and chelates Mg^2+^, thus revealing the already observed accommodation [6] at the IN active site (Figure 6).

Moreover, compounds **2h**, **3c** and **3d**, showed similar free binding energy, when compared with the reference compound **III** (∆Gbind = -6.86, -6.55, -6.56 and -6.78 kcal/mol for **2h**, **3c**, **3d** and **III**, respectively). Interestingly, the three keto-enol tautomers of **3a** shared different free binding energy results (∆Gbind = -4.41, -5.56, and -5.61 kcal/mol for **3a_1_**, **3a_2_**, and **3a_3_**, respectively). In fact, while **3a_1_** showed a significantly lower binding energy, for **3a_2_**, and **3a_3_** a similar behaviour in terms of both free binding energy and binding modes was observed. This could be due to the fact that the keto-enolic tautomers could potentially be able to establish a coordination bond with Mg^2+^ ion through an hydroxy-keto system (for **3a_2_**) or to strongly bind (for **3a_3_**) with important residues on the active site of IN as displayed in Figure 7.

## Conclusions

We have synthesized novel symmetric and asymmetric bis-amides and hydrazides derivatives of malonic acid as prototypes of IN inhibitors and built a unified pharmacophore to help us in rational design of future inhibitors. Docking studies were performed on title and reference compounds to predict their binding interaction with the active site of IN. Studies are in progress to optimize their potency and physicochemical properties.

## Experimental

### General

Anhydrous solvents and all reagents were purchased from Sigma-Aldrich, Merck, Acros or Carlo Erba. Reactions involving air- or moisture-sensitive compounds were performed under a nitrogen atmosphere using oven-dried glassware and syringes to transfer solutions. Melting points (m.p.) were determined using an Electrothermal melting point or a Köfler apparatus and are uncorrected. Infrared (IR) spectra were recorded as thin films or nujol mulls on NaCl plates with a Perkin-Elmer 781 IR or 983 spectrophotometers and are expressed in ν (cm^-1^). Nuclear magnetic resonance spectra (^1^H-NMR and ^13^C-NMR) were determined in CDCl_3_/DMSO-*d_6_* (in 3/1 ratio) or DMSO-*d_6_* and were recorded on a Varian XL-200 (200 MHz) or a Varian VXR-300 (300 MHz). Chemical shifts (δ scale) are reported in parts per million (ppm) downfield from tetramethylsilane (TMS) used as internal standard. Splitting patterns are designated as follows: s, singlet; d, doublet; t, triplet; q, quadruplet; m, multiplet; brs, broad singlet; dd, double doublet. The assignment of exchangeable protons (O*H* and N*H*) was confirmed by addition of D_2_O. Analytical thin-layer chromatography (TLC) was carried out on Merck silica gel F-254 plates. For flash chromatography Merck Silica gel 60 was used as stationary phase with a particle size 0.040-0.063 mm (230-400 mesh ASTM). Elemental analyses were performed on a Perkin-Elmer 2400 spectrometer, and were within ±0.4% of the theoretical values.

### General procedure for the synthesis of symmetric bis-amides **(1a-h)**

Dimethyl malonate (for **1a-e** and **h**) or diethyl malonate (for **1f-g**) (5.6 mmols) and the corresponding aromatic amine (11.0 mmols) were mixed under an argon atmosphere in a 25 mL two-neck round-bottom flask fitted with a reflux condenser and a magnetic bar. The mixture was stirred at 185 °C for 3.5-5 h. The solids obtained were triturated from EtOH and isolated by filtration. The products were characterized as reported below.

*N,N’-Diphenylmalonamide* (**1a**). White solid, crystallized from EtOH. Yield = 72 %; m.p. = 228 – 230 °C (lit. [12] 228 - 229 °C); IR ν cm^-1^ = 3220 (NH); 1650 (C=O); ^1^H-NMR (DMSO-*d*_6_, 80 °C) δ 10.06 (s, 2H, NH), 7.60 (d, 4H, *J =*8.4 Hz, Ar-H), 7.28-7.34 (m, 4H, Ar-H), 7.06 (t, 2H, *J =*7.5 Hz, Ar-H), 3.48 (s, 2H, CH_2_); ^13^C-NMR (DMSO-*d*_6_, 80 °C) δ 164.9, 138.5, 128.2, 123.0, 119.1, 118.9, 45.3; Anal. Calcd. for C_15_H_14_N_2_O_2_: C, 70.85; H, 5.56; N, 11.01. Found: C, 71.32; H, 5.63; N, 10.98.

*N,N’-bis-(2-Methoxyphenyl)malonamide* (**1b**)*.* White needle-like crystals, crystallized from EtOH. Yield = 34 %; m.p. = 163 °C (lit. [19] 159 - 160 °C); IR ν cm^-1^ = 3310 (NH); 1645 (C=O); ^1^H-NMR (DMSO-*d*_6_, 50 °C) δ 9.62 (brs, 2H, NH), 8.02 (d, 2H, *J =*9 Hz, Ar-H), 7.03-7.09 (m, 4H, Ar-H), 6.86-6.95 (m, 2H, Ar-H), 3.83 (s, 6H, OCH_3_), 3.70 (s, 2H, CH_2_). Anal. Calcd. for C_17_H_18_N_2_O_4_: C, 64.96; H, 5.77; N, 8.91. Found: C, 64.94; H, 5.75; N, 8.89.

*N,N’-bis-(3-Methoxyphenyl)malonamide* (**1c**)*.* White needle-like crystals, crystallized from DMSO/H_2_O. Yield = 32 %; m.p. = 146 °C (lit. [32] 158 - 160 °C); IR (nujol) ν cm^-1^ = 3250 (NH); 1600 (C=O); ^1^H-NMR (DMSO-*d*_6_, 50 °C) δ 10.15 (s, 2H, NH), 7.31 (t, 2H, *J =*2.1 Hz, Ar-H), 7.21 (t, 2H, *J =*8.1 Hz, Ar-H), 7.10-7.13 (m, 2H, Ar-H), 6.56 (dd, 2H, *J =*0.9, 3.5 Hz, Ar-H), 3.73 (s, 6H, OCH_3_), 3.45 (s, 2H, CH_2_); ^13^C-NMR (DMSO-*d*_6_) δ 165.5, 159.6, 140.2, 129.7, 111.4, 108.9, 104.9, 55.0, 46.1. Anal. Calcd. for C_17_H_18_N_2_O_4_: C, 64.95; H, 5.78; N, 8.90. Found: C, 64.93; H, 6.12; N, 8.82.

*N,N’-bis-(4-Methoxyphenyl)malonamide* (**1d**)*.* White solid, crystallized from EtOH. Yield = 20 %; m.p. = 200 °C (lit. [12] 232 - 234 °C); IR (nujol) ν cm^-1^ = 3240 (NH); 1620 (C=O); ^1^H-NMR (DMSO-*d*_6_, 50°C) δ 9.91 (s, 2H, NH), 7.49-7.52 (m, 4H, Ar-H), 6.87-6.90 (m, 4H, Ar-H), 3.73 (s, 6H, OCH_3_), 3.41 (s, 2H, CH_2_); ^13^C-NMR (DMSO-*d*_6_) δ 165.0, 155.3, 132.2, 120.6, 113.9, 55.1, 45.6; Anal. Calcd. for C_17_H_18_N_2_O_4_: C, 64.95; H, 5.78; N, 8.90. Found: C, 65.38; H, 6.03; N, 8.90.

*N,N’-bis-(3-Chlorophenyl)malonamide* (**1e**)*.* White solid, crystallized from EtOH. Yield = 29 %; m.p. = 162 °C (lit. [33] 165 - 166 °C); IR (nujol) ν cm^-1^ = 3225 (NH); 1620 (C=O); ^1^H-NMR (DMSO-*d*_6_, 80 °C) δ 10.14 (brs, 2H, NH), 7.79 (t, 2H, *J =*1.8 Hz, Ar-H), 7.44-7.48 (m, 2H, Ar-H), 7.34 (t, 2H, *J* = 8.1, Hz, Ar-H), 7.09-7.13 (m, 2H, Ar-H), 3.50 (s, 2H, CH_2_); ^13^C-NMR (DMSO-*d*_6_, 80 °C) 165.0, 139.0, 133.0, 130.0, 123.0, 118.0, 117.0, 45.0; Anal. Calcd. for C_15_H_12_Cl_2_N_2_O_2_: C, 55.75; H, 3.74; N, 8.67. Found: C, 55.63; H, 3.64; N, 8.60.

*N,N'-bis-(4-Chlorophenyl)malonamide* (**1f**). White solid, crystallized from EtOH. Yield = 40 %; m.p. = 261 °C (lit. [12] 256 - 257 °C); IR (nujol) ν cm^-1^ = 3210 (NH); 1620 (C=O); ^1^H-NMR (DMSO-*d*_6_) δ 10.34 (s, 2H, NH), 7.63 (d, 4H, *J =*9 Hz, Ar-H), 7.35 (d, 4H, *J =*8.7, Hz, Ar-H), 3.48 (s, 2H, CH_2_); ^13^C-NMR (DMSO-*d*_6_) δ 165.0, 138.0, 129.0, 127.0, 120.0, 46.0; Anal. Calcd. for C_15_H_12_Cl_2_N_2_O_2_: C, 55.75; H, 3.74; N, 8.67. Found: C, 55.94; H, 3.67; N, 8.74.

*Preparation of N,N’-diquinol-3-yl-malonamide* (**1g**)*.* In a 25 mL round-bottom flask fitted with a reflux condenser and a stirring bar were introduced, under an argon atmosphere, 0.50 g (3.4 mmols) of 3-aminoquinoline and 0.27 g (1.7 mmols) of diethylmalonate in 7 mL of anhydrous DMF and the mixture was refluxed for 8 h. Then the reaction was cooled down to r.t., quenched with 10 ml of H_2_O and stirred at r.t. for few minutes. The solid formed was filtered through a Büchner and then was washed with Et2O and acetone. A white solid was obtained 0.37 g (1.0 mmol) that was purified by crystallization from DMSO/H_2_O. Yield = 46 %; m.p. = 160 - 162 °C; IR (nujol) ν cm^-1^ = 3140 (NH); 1660 (C=O); ^1^H-NMR (DMSO-*d*_6_, 50 °C) δ 10.43 (s, 2H, NH), 8.89 (d, 2H, *J =*3.0, Hz, Ar-H), 8.72 (d, 2H, *J =*17.4 Hz, Ar-H), 7.89-7.98 (m, 4H, Ar-H), 7.52-7.67 (m, 4H, Ar-H), 3.40 (s, 2H, CH_2_); ^13^C-NMR (DMSO-*d*_6_) δ 169.2, 166.1, 144.4, 144.3, 144.1, 133.0, 128.0, 127.8, 127.7, 127.6, 127.0, 121.0, 24.0; Anal. Calcd. for C_21_H_16_N_4_O_2_: C, 70.77; H, 4.53; N, 15.72. Found: C, 70.53; H, 5.60; N, 14.86.

*N,N’-Diquinol-8-yl-malonamide* (**1h**)*.* White solid, crystallized from DMSO/H_2_O. Yield = 37 %; m.p. = 214 °C (lit. [34] 218 - 219 °C); IR (nujol) ν cm^-1^ = 3240 (NH); 1620 (C=O); ^1^H-NMR (DMSO-*d*_6_, 50 °C) δ 10.70 (s, 2H, NH), 8.93 (dd, 2H, *J =*4.0, 1.5 Hz, Ar-H), 6.67 (d, 2H, *J =*7.5 Hz, Ar-H), 8.39 (dd, 2H, *J =*9.0, 1.5 Hz, Ar-H), 7.56-7.69 (m, 4H, Ar-H), 4.07 (s, 2H, CH_2_); ^13^C-NMR (DMSO-*d*_6_) δ 165.5, 148.5, 138.1, 136.0, 134.0, 127.5, 126.4, 121.8, 121.6, 116.7, 45.6; Anal. Calcd. for C_21_H_16_N_4_O_2_·0.5H_2_O: C, 69.03; H, 4.69; N, 15.33. Found: C, 69.31; H, 4.32; N, 15.14.

*General procedure for the synthesis of asymmetric bis-amides* **2b-h**. In a two-neck 50 mL round-bottom flask were introduced, under an argon atmosphere, an aromatic amine (2.6 mmols) and the monophenylamide of malonic acid **7** (0.46 g, 2.6 mmols) in anhydrous THF (20 mL). The reaction mixture was stirred at r.t. for 15 minutes. Then, 4-(4,6-dimethoxy-[1,3,5]-triazyn-2-yl)-4-methyl-morpholine chloride (DMTMM, 0.70 g, 2.6 mmols) were added and the solution was stirred at r.t. for 24 h. The solvent was evaporated under *vacuo* and the solid residue obtained was washed with water, an aqueous saturated solution of NaHCO_3_, a 1.0 M aqueous solution of HCl and brine. Finally, the solid was washed with diethyl ether and filtered.

*N-(2-Methoxyphenyl)-N’-phenylmalonamide* (**2b**). [35] White solid, crystallized from DMSO/H_2_O. Yield = 25 %; m.p. = 154 - 155 °C; IR (nujol) ν cm^-1^ = 3230 (NH); 1650 (C=O); ^1^H-NMR (DMSO-*d*_6_) δ 10.24 (s, 1H, NH), 9.68 (brs, 1H, NH), 8.09 (d, 1H, *J =*8.1 Hz, Ar-H), 7.60 (d, 2H, *J =*7.8 Hz, Ar-H), 7.32 (t, 2H, *J =*7.8 Hz, Ar-H), 7.05-7.09 (m, 3H, Ar-H), 6.91-6.93 (m, 1H, Ar-H), 3.85 (s, 3H, OCH_3_); ^13^C-NMR (DMSO-*d*_6_) δ 166.1, 165.1, 148.9, 138.7, 128.8, 127.2, 124.2, 123.5, 120.7, 120.3, 119.2, 111.0, 55.7, 45.1; Anal. Calcd. for C_16_H_16_N_2_O_3_·0.5H_2_O: C, 65.52; H, 5.84; N, 9.55. Found: C, 65.15; H, 5.83; N, 9.77.

*N-(3-Methoxyphenyl)-N’-phenylmalonamide* (**2c**). White solid, crystallized from DMSO/H_2_O. Yield = 26 %; m.p. = 144 - 145 °C; IR (nujol) ν cm^-1^ = 3520 (NH); 1630 (C=O); ^1^H-NMR (DMSO-*d*_6_) δ 10.2 (brs, 2H, NH), 7.60 (d, 2H, *J =*8.1 Hz, Ar-H), 7.31 (t, 3H, *J =*7.2 Hz, Ar-H), 7.21 (t, 1H, *J =*8.1 Hz, Ar-H), 7.12 (d, 1H, *J =*8.1 Hz, Ar-H), 7.05 (t, 1H, *J =*7.5 Hz, Ar-H), 6.63-6.65 (m, 1H, Ar-H), 3.72 (s, 3H, OCH_3_), 3.46 (s, 2H, CH_2_); ^13^C-NMR (DMSO-*d*_6_) δ 165.5, 165.3, 159.5, 140.1, 139.0, 129.5, 128.7, 123.3, 119.0, 11.3, 108.8, 104.8, 54.9, 46.0; Anal. Calcd. for C_16_H_16_N_2_O_3_·0.25H_2_O: C, 66.54; H, 5.76; N, 9.70. Found: C, 66.80; H, 5.44; N, 9.75.

*N-(4-Methoxyphenyl)-N’-phenylmalonamide* (**2d**). [36] White solid, crystallized from DMSO/H_2_O followed by crystallisation from THF/petrol ether. Yield = 18 %; m.p. = 186 °C; IR (nujol) ν cm^-1^ = 3250 (NH); 1620 (C=O); ^1^H-NMR (DMSO-*d*_6_) δ 9.92 (brs, 2H, NH), 9.79 (brs, 1H, NH), 7.42-7.61 (m, 4H, Ar-H), 7.31 (t, 2H, *J =*7.2 Hz, Ar-H), 7.06 (t, 1H, *J =*6.9 Hz, Ar-H), 6.89 (d, 2H, *J =*8.1 Hz, Ar-H, 3.74 (s, 3H, OCH_3_), 3.46 (s, 2H, CH_2_); ^13^C-NMR (DMSO-*d*_6_) δ 165.5, 164.5.3, 155.2, 138.5, 131.7, 128.2, 123, 120.6, 119, 113.6, 54.9, 45.1; Anal. Calcd. for C_16_H_16_N_2_O_3_·0.25H_2_O: C, 66.54; H 5.76; N, 9.70. Found: C, 66.08; H, 5.57; N, 10.04.

*N-(3-Chlorophenyl)-N’-phenylmalonamide* (**2e**). White solid, crystallized from THF/petrol ether. Yield = 28 %; m.p. = 167 °C; IR (nujol) ν cm^-1^ = 3230 (NH); 1630 (C=O); ^1^H-NMR (DMSO-*d*_6_) δ 10.41 (s, 1H, NH), 10.21 (s, 1H, NH), 7.84 (s, 1H, Ar-H), 7.60 (d, 2H, *J =*8.4 Hz, Ar-H), 7.42 (d, 1H, *J =*8.1 Hz, Ar-H), 7.29-7.37 (m, 3H, Ar-H), 7.12 (d, 1H, *J =*7.8 Hz, Ar-H), 7.06 (t, 1H, *J =*6.6 Hz, Ar-H), 3.48 (s, 2H, CH_2_); ^13^C-NMR (DMSO-*d*_6_) δ 165.8, 165.1, 140.4, 138.9, 133.1, 130.5, 128.9, 123.4, 123.1, 119.0, 118.5, 117.4, 46.0; Anal. Calcd. for C_15_H_13_ClN_2_O_2_: C, 62.40; H, 4.54; N, 9.70. Found: C, 62.53; H, 4.57; N, 9.63.

*N-(4-Chlorophenyl)-N’-phenylmalonamide* (**2f**). White solid, crystallized from THF/petrol ether. Yield = 32 %; m.p. = 220 °C; IR (nujol) ν cm^-1^ = 3220 (NH); 1630 (C=O); ^1^H-NMR (DMSO-*d*_6_) δ 10.18 (brs, 1H, NH), 10.02 (brs, 1H, NH), 7.62 (t, 4H, *J =*9 Hz, Ar-H), 7.33 (t, 4H, *J =*8.4 Hz, Ar-H), 7.05-7.09 (m, 1H, Ar-H), 3.50 (s, 2H, CH_2_); ^13^C-NMR (DMSO-*d*_6_) δ 165.1, 164.7, 138.5, 137.4, 128.18, 128.1, 126.7, 122.9, 120.5, 119.0, 45.3, 40.3; Anal. Calcd. for C_16_H_13_ClN_2_O_2_: C, 62.40; H, 4.54; N, 9.70. Found: C, 62.31; H, 4.57; N, 9.36.

*N-Phenyl-N’-quinol-3-yl-malonamide* (**2g**). White solid, crystallized from DMSO/H_2_O. Yield = 17 %; m.p. = 200 - 202 °C; IR (nujol) ν cm^-1^ = 3230 (NH); 1630 (C=O); ^1^H-NMR (DMSO-*d*_6_) δ 11.27 (s, 1H, NH), 10.45 (s, 1H, NH), 9.20 (s, 1H, Ar-H), 8.95 (s, 1H, Ar-H), 8-8.1 (m, 2H, Ar-H), 7.79 (t, 1H, *J =*7.5 Hz, Ar-H), 7.6-7.7 (m, 3H, Ar-H), 7.31 (t, 2H, *J =*8.1 Hz, Ar-H), 7.05 (t, 1H, *J =*7.2 Hz, Ar-H) 3.67 (s, 2H, CH_2_); ^13^C-NMR (DMSO-*d*_6_) δ 166.6, 164.9, 141.6, 140.1, 138.9, 133.1, 129.9, 128.7, 128.3, 128.2, 128.1, 126, 125.2, 123.4, 119.1, 45.9; Anal. Calcd. for C_18_H_15_N_3_O_2_: C, 70.81; H, 4.95; N, 13.76. Found: C, 69.93; H, 5.04; N, 13.45.

*N-Phenyl-N’-quinol-8-yl-malonamide* (**2h**)*.* Light brown solid, crystallized from DMSO/H_2_O. Yield = 24 %; m.p. = 184 - 185 °C; IR (nujol) ν cm^-1^ = 3260 (NH); 1650 (C=O); ^1^H-NMR (DMSO-*d*_6_, 50 °C) δ 10.86 (brs, 1H, NH), 10.24 (brs, 1H, NH), 8.94 (brs, 1H, Ar-H), 8.67 (brs, 1H, Ar-H), 8.39 (brs, 1H, Ar-H), 7.58-7.64 (m, 5H, Ar-H), 7.20-7.40 (m, 2H, Ar-H), 7.0-7.20 (m, 1H, Ar-H), 3.78 (s, 2H, CH_2_); ^13^C-NMR (DMSO-*d*_6_, 50 °C) δ 165.7, 165.2, 148.7, 138.5, 138.0, 136.2, 134.3, 128.5, 127.6, 123.4, 121.8, 121.8, 119.3, 116.3, 45.6; Anal. Calcd. for C_18_H_15_N_3_O_2_: C, 70.81; H, 4.95; N, 13.76. Found: C, 71.23; H, 5.48; N, 13.63.

### Preparation of N-phenylmalonamic acid ***(7)***.

*Method a)* In a two-neck 100 mL round-bottom flask fitted with a condenser and stir bar were introduced under an argon atmosphere aniline **4a** (6.5 g, 7.0 mmols) and dimethylmalonate (13.8 g, 10.0 mmols). The reaction mixture was vigorously stirred at 190 °C for 30 minutes and then cooled to r.t. and diluted with ethanol (40 mL). The solid formed was filtered through Büchner and the filtrate was diluted with water (50 mL) in order to facilitate the precipitation of diamide still in solution that was separated by filtration. The filtrate was extracted with dichloromethane (DCM) and the combined organic layers were washed with 1N HCl, dried over Na_2_SO_4_, filtered and the solvent was evaporated *in vacuo* to give a yellow oil (5.08 g, 2.6 mmols, 37 % yield); TLC (ethyl acetate/petroleum ether = 7/3). The obtained compound was transferred in a 250 mL round bottom flask fitted with a condenser and a stirring bar and was suspended in a solution of Na_2_CO_3_ (5.29 g, 5.0 mmols) in water (100 mL). The mixture was refluxed for 30 min, cooled down with a slush bath and acidified by slow addition of 37 % HCl until a white precipitate was formed. The solid was collected by filtration and washed with 0.2N HCl and diethyl ether. Yield = 13 %; m.p. = 132 °C (lit. [15] 132 °C); IR (nujol) ν cm^-1^ = 1710 (C=O), 1660 (C=O); ^1^H-NMR (DMSO-*d*_6_, 80°C) δ 9.98 (brs, 1H, NH), 7.57 (d, 2H, *J =*8.1 Hz, Ar-H), 7.29 (t, 2H, *J =*7.5 Hz, Ar-H), 7.05 (t, 1H, *J =*7.5 Hz, Ar-H), 3.36 (s, 2H, CH_2_); ^13^C-NMR (DMSO-*d*_6_, 80°C) δ 164.9, 138.5, 128.2, 123.0, 119.1, 118.9, 45.3; Anal. Calcd. For C_9_H_9_NO_3_: C, 60.33; H, 5.06; N, 7.82. Found: C, 60.31; H, 5.04; N, 7.80.

*Method b).* To a two-neck 500 mL round bottom flask fitted with a condenser and a stirring bar containing anhydrous THF (150 mL) were added under a nitrogen atmosphere monobenzyl ester **9** (6.0 g, 30.0 mmols) and aniline **4a** (2.73 mL, 30.0 mmol). The solution was stirred at r.t. for 15 min. and then DMTMM (8.3 g, 30.0 mmols) dissolved in anhydrous THF (50 mL) were added. The reaction mixture was stirred at r.t. for 24 h and then the solvent was evaporated *in vacuo* to give a white residue that was dissolved in DCM, washed with a saturated aqueous solution of NaHCO_3_, water, 1N HCl and brine. The organic layer was dried over Na_2_SO_4_, filtered and the solvent was evaporated *in vacuo* to afford **10** as a white solid that was triturated from petroleum ether and DCM (5.2 g, 52.4 mmols). Yield = 62 %); m.p. = 62 - 63 °C.

The amido ester **10** was transferred under a nitrogen atmosphere into a 100 mL two-neck flask fitted with a stirring bar containing anhydrous MeOH (50 mL). Then 5 % Pd/C (0.52 g, 0.2 mmols of Pd/0.01 eq) was added and the mixture was hydrogenated at r.t. and atmospheric pressure for 4 h. A second portion of 5 % Pd/C (0.52 g, 0.2 mmols of Pd/0.01 eq) was added and the reaction stirred for 12 h. The mixture was treated with nitrogen, filtered, and the filtrated was evaporated *in vacuo* to give **7** as a white solid (2.96 g, 16.5 mmols). Yield = 86 %; m.p. = 132 °C (lit. [15] 132 °C).

*Synthesis of 3-hydrazyn-3-oxo-N-phenylpropanamide* (**3a**). Hydrazine monohydrate **12a** (0.20 mL, 4.12 mmols) was added to a solution of **11** (0.30 g, 1.45 mmols) in absolute ethanol (10 mL) and the reaction was stirred at reflux for 4 h. The mixture was cooled to r.t. and the white crystals formed were filtered and washed with ethanol. Yield = 59 %; m.p. = 177 - 179 °C (lit. [37] 183 °C); IR (nujol) ν cm^- 1^ = 1667 (C=O), 1640 (C=O); ^1^H-NMR (DMSO-*d*_6_) δ 10.09 (s, 1H, NH, exchange with D_2_O), 9.19 (s, 1H, NH, exchange with D_2_O), 7.58 (d, 2H, Ar-H), 7.30 (t, 2H, Ar-H), 7.05 (t, 1H, Ar-H), 4.30 (s, 2H, NH_2_ exchange with D_2_O), 3.18 (s, 2H, CH_2_); ^13^C-NMR (DMSO-*d*_6_) δ 166.1, 165.4, 138.9, 128.6, 123.2, 119.1, 42.9; GC/MS: *m/z* 193 (M^+^); Anal. Calcd. for C_9_H_11_N_3_O_2_: C, 55.95; H, 5.74; N, 21.75. Found: C, 56.29; H, 6.02; N, 22.03.

*Synthesis of 3-(2-methyhydrazin)-3-oxo-N-phenylpropanamide* (**3b**). Methylhydrazine monohydrate **12b** (0.26 mL, 4.83 mmols) was added to a solution of **11** (0.40 g, 1.93 mmols) in absolute ethanol (10 mL) and the reaction was stirred at reflux for 30 h. The mixture was cooled down to r.t. and the white crystals formed were filtered. Yield = 50 %; m.p. = 169 - 171 °C; IR (nujol) ν cm^-1^ = 1666 (C=O), 1636 (C=O); ^1^H-NMR (CDCl_3_ + DMSO-*d*_6_) δ 10.05 (s, 1H, NH, exchange with D_2_O), 9.49 (s, 1H, NH exchange with D_2_O), 7.59 (d, 2H, Ar-H), 7.28 (t, 2H, Ar-H), 7.03 (t, 1H, Ar-H), 4.71 (s, 1H, NH), 3.21 (s, 2H, CH_2_), 2.50 (s, 3H, CH_3_); ^13^C-NMR (CDCl_3_ + DMSO-*d*_6_) δ 163.7, 163.4, 137.1, 128.7, 121.5, 117.3, 41.2, 36.8; GC/MS: *m/z* 207 (M^+^); Anal. Calcd. for C_10_H_13_N_3_O_2_: C, 57.96; H, 6.32; N, 20.28. Found: C, 58.20; H, 6.60; N, 20.50.

*Synthesis of 3-oxo-N-phenyl-3-(2-phenylhydrazin)propanamide* **(3c)** *and N-N^1^-diphenylmalonamide **(1a)**.* A solution of **11** (0.40 g, 1.93 mmols) and phenylhydrazine **12c** (0.27 mL, 2.70 mmols) in chlorobenzene (14 mL) was stirred at reflux for 20 h. Then, the solvent was removed *in vacuo* to give a brown residue that was purified by silica gel column chromatography eluting with ethyl acetate/petroleum ether 7.5/2.5 to afford **3c** and **1a**. (**3c**): Yield = 31 %; m.p. = 173 - 175 °C; IR (nujol) ν cm^-1^ = 1668 (C=O), 1642 (C=O); ^1^H-NMR (CDCl_3_ + DMSO-*d*_6_) δ 10.10 (s, 1H, NH, exchange with D_2_O), 9.97 (s, 1H, NH, exchange with D_2_O), 9.83 (s, 1H, NH, exchange with D_2_O), 7.72-7.59 (m, 2H, Ar-H), 7.32-7.04 (m, 5H, Ar-H), 6.82-6.70 (m, 3H, Ar-H), 3.38 (s, 2H, CH_2_); C-NMR (CDCl_3_ + DMSO-*d*_6_) δ 166.4, 165.1, 148.8, 138.8, 128.4, 123.2, 119.0, 118.5, 112.2, 43.0; GC/MS: *m/z* 269 (M^+^); Anal. Calcd. for C_15_H_15_N_3_O_2_: C, 66.90; H, 5.61; N, 15.60. Found: C, 67.15; H, 5.89; N, 15.82. (**1a**): Yield = 23 %; m.p. = 227 - 228 °C (lit. 228 - 229 °C) [7]; IR (nujol) ν cm^-1^ = 1650 (C=O); ^1^H-NMR (CDCl_3_+ DMSO-*d*_6_) δ 10.10 (s, 2H, NH), 7.62 (d, 4H, Ar-H), 7.28 (t, 4H, Ar-H), 7.04 (t, 2H, Ar-H), 3.50 (s, 2H, CH_2_); C-NMR (CDCl_3_+DMSO-*d*_6_) δ 164.6, 137.4, 127.6, 122.7, 118.6, 44.1; GC/MS: *m/z* 254 (M^+^); Anal. Calcd. for C_15_H_14_N_2_O_2_: C, 70.85; H, 5.55; N, 11.02. Found: C, 71.00; H, 5.73; N, 11.29.

*Synthesis of 3-[2-(4-nitrophenylhydrazin]-3-oxo-N-phenylpropanamide* (**3d**). A mixture of **11** (0.40 g, 1.93 mmols) and 4-nitrophenylhydrazine **12d** (0.44 g, 2.9 mmols) in chlorobenzene (20 mL) was stirred at reflux for 15 h. Then, the solvent was removed in vacuo to give a pale orange solid that was triturated with diethyl ether and petroleum ether followed by silica gel column chromatography eluting with ethyl acetate/petroleum ether 6/4. Yield = 63 %; m.p. = 205 - 207 °C; IR (nujol) ν cm^-1^ = 1668 (C=O), 1642 (C=O); ^1^H-NMR (CDCl_3_ + DMSO-*d_6_*) δ 10.06 (s, 1H, NH, exchange with D_2_O), 9.91 (s, 1H, NH, exchange with D_2_O), 8.59 (s, 1H, NH, exchange with D_2_O), 8.05 (d, 2H, J =9, Ar-H), 7.73- 7.50 (m, 3H, Ar-H), 7.30 (t, 1H, Ar-H), 7.08 (t, 1H, Ar-H), 6.88 (d, 2H, J = 9, Ar-H), 3.48 (s, 2H, CH_2_); ^13^C-NMR (CDCl_3_ + DMSO-*d_6_*) δ 165.9, 163.8, 153.3, 137.7, 137.4, 129.8, 127.4, 124.4, 122.5, 118.4, 109.7, 41.9; GC/MS: m/z 314 (M^+^); Anal. Calcd. for C_15_H_14_N_4_O_4_: C, 57.32; H, 4.49; N, 17.83. Found: C, 57.61; H, 4.73; N, 18.10.

*Synthesis of 3-oxo-N-phenyl-3-{2-[4-(trifluoromethyl)pyrimidin-2-yl]hydrazin}propanamide (**3e**) and N^1^,N^3^-bis[4-(trifluoromethyl)pyrimidin-2-yl]malonylhydrazide* (**14**). 4-Trifluoromethyl-2-hydrazino-pyrimidine **12e** (0.45 g, 2.53 mmols) was added to a solution of **11** (0.35 g, 1.69 mmols) in chlorobenzene (15 mL) and the mixture was stirred at reflux for 25 h. The solution was cooled down to r.t. and the precipitate formed was filtered and washed with chlorobenzene. Then, the solid was washed with ethyl acetate to give **14** as a white solid that remained on the filter, and evaporation of the organic layers *in vacuo* afforded **3a** as a yellow solid. (**14**) Yield = 60 %; m.p. = 245 - 247 °C; IR (nujol) ν cm^-1^ = 1650 (C=O), 1152 (CF); ^1^H-NMR (DMSO-d_6_) δ 10.13-9.60 (2 brs, 4H, NH), 8.72 (d, 2H, Ar-H), 7.21 (d, 2H, Ar-H), 3.27 (s, 2H, CH_2_); ^13^C-NMR (DMSO-d_6_) δ 165.3, 162.6, 161.4, 122.9, 117.4, 107.0, 42.5; GC/MS: m/z 424 (M^+^); Anal. Calcd. for C_13_H_10_F_6_N_8_O_2_: C, 36.80; H, 2.38; N, 26.41. Found: C, 37.10; H, 2.64; N, 26.20. (**3e**) Yield = 21 %; m.p. = 167 - 169 °C; IR (nujol) ν cm^-1^ = 1688 (C=O), 1668 (C=O), 1153 (CF); ^1^H-NMR (DMSO-d_6_) δ 10.18 (s, 1H, NH), 10.14 (s, 1H, NH), 9.74 (s, 1H, NH), 8.73 (d, 1H, Ar-H), 7.60 (d, 1H, Ar-H), 7.45-7.24 (m, 4H, Ar-H), 7.21 (d, 1H, Ar-H), 7.06 (t, 1H, Ar-H); ^13^C-NMR (DMSO-d_6_) δ 166.5, 165.0, 163.2, 162.0, 139.0, 130.4, 128.9, 128.5, 127.2, 123.7, 123.4, 119.3, 117.9, 107.7; GC/MS: m/z 339 (M^+^); Anal. Calcd. for C_14_H_12_F_3_N_5_O_2_: C, 49.56; H, 3.57; N, 20.64. Found: C, 51.10; H, 4.16; N, 20.80.

*Synthesis of 3-[2-(3,4-dihydronaphtalen-1(2H)-hylidene)hydrazin]-3-oxo-N-phenylpropanamide* (**3f**)*.* Tetralone **13** (1.0 mL, 1.1 g, 7.5 mmols) was added dropwise to a solution of hydrazide **3a** (0.40 g, 2.1 mmols) in absolute ethanol (50 mL), which was obtained by heating the mixture at 60 °C. Then, the reaction was stirred at reflux for 5 h until a white solid appeared. The residue formed was filtered and washed with ethanol. Yield = 84 %; m.p. = 234 - 235 °C; IR (nujol) ν cm^-1^ = 1682 (C=O), 1654 (C=O); ^1^H-NMR (DMSO-*d*_6_) δ 10.64 (s, 1H, NH), 10.21 (s, 1H, NH), 7.99 (d, 1H, Ar-H), 7.62 (d, 2H, Ar-H), 7.40-6.95 (m, 6H, Ar-H), 3.75 (s, 2H, CH_2_), 2.82-2.54 (m, 4H, CH_2_), 1.90-1.69 (m, 2H, CH_2_); ^13^C-NMR (DMSO-*d*_6_) δ 169.9, 165.7, 151.4, 147.1, 132.2, 128.8, 128.7, 128.5, 126.1, 124.5, 123.1, 119.0, 43.9, 28.8, 25.6, 21.3; GC/MS: m/z 321 (M^+^); Anal. Calcd. for C_19_H_19_N_3_O_2_: C, 71.01; H, 5.96; N, 13.08. Found: C, 71.28; H, 6.10; N, 13.20.

*Synthesis of ethyl-3-anilin-3-oxopropanoate* (**11**) [15]. 2.0 g (1.70 mL, 13.3 mmols) of ethyl malonyl chloride (EMC) was added to a solution of aniline **4a** (1.12 g, 1.1 mL, 12.1 mmols) and triethylamine (TEA) (1.85 mL, 13.3 mmols) in acetone (50 mL). The reaction mixture was stirred at r.t. for 1h and the solvent was evaporated *in vacuo* to give a residue that was suspended in water, treated with 3N HCl and extracted with diethyl ether. The combined organic layers were dried over Na_2_SO_4_, filtered and the solvent was evaporated *in vacuo* to give an orange oil. Yield = 89 %; m.p. = oil at r.t.; IR (nujol) ν cm^-1^ = 1744 (C=O ester); 1669 (C=O amide); ^1^H-NMR (DMSO-*d*_6_) δ 10.18 (s, 1H, NH, exchange with D_2_O), 7.58 (d, 2H, Ar-H), 7.31 (t, 2H, Ar-H), 7.06 (t, 1H, Ar-H), 4.12 (q, 2H, OCH_2_CH_3_), 3.46 (s, 2H, CH_2_), 1.21 (t, 3H, OCH_2_CH_3_); ^13^C-NMR (DMSO-*d*_6_) δ 167.7, 164.1, 138.8, 128.8, 123.5, 119.1, 60.7, 43.7, 14.0; GC/MS: *m/z* 207 (M^+^); Anal. Calcd. for C_11_H_13_NO_3_: C, 63.76; H, 6.32; N, 6.76. Found: C, 63.94; H, 6.65; N, 6.90.

### Molecular Modeling

Model compounds **I-Phe-DKA**, **III**, **2a**, **2h**, **3a_1_**, **3a_2_**, **3a_3_**, **3c**, **3d**, and **3e** were constructed with standard bond lengths and angles from the fragment database with MacroModel 6.0 [38] using a Silicon Graphics O2 workstation running on IRIX 6.3. Sybyl 6.2 (2001) [39] was used as graphic platform. The atomic charges were assigned using the Gasteiger-Marsili method [40]. Minimization of structures was performed with the MacroModel/BachMin 6.0 program using the AMBER force field. Extensive conformational search was carried out using the Monte Carlo/Energy minimization [41] for all the compounds considered in the study (E_i_-E_min_ < 5 Kcal/mol, energy difference between the generated conformation and the current minimum). Final minimization of the structures was performed with Sybyl 6.2 by using Tripos force field. Docking calculations were performed on Linux workstation.

Subunit A of IN core domain in complex with 1-(5-chloroindol-3-yl)-3-hydroxy-3-(2*H*-tetrazol-5-yl-propenone) (5CITEP; PDB 1QS4) was used for all docking studies. The missing residues at positions 141-144 in this subunit were incorporated from monomer B of the IN structure PDB 1BIS after superimposition of the backbones of residues 135-140 and 145-150, as previously reported [21,22,23]. The atomic charges for the protein were assigned using the Kollman United method [42]. Docking was performed with AutoDock version 3.05 [28] using the empirical free energy function and the Lamarckian protocol [43]. Mass-centered grid maps were generated with 80 grid points for every direction and with 0.375 Angstroms spacing by the AutoGrid program for the whole protein target.

Random starting position on the entire protein surface, random orientations and torsions were used for the ligands. The distance-dependent dielectric permittivity of Mehler and Solmajer was used for the calculation of the electrostatic grid-maps. 100 independent docking runs were carried out for each ligands. The cluster analyses were computed with a cluster tolerance by less than 1.5 Å in positional root-mean-square deviation with AutoDock Tools 1.4.6 [44].
